# PF-4var/CXCL4L1 Predicts Outcome in Stable Coronary Artery Disease Patients with Preserved Left Ventricular Function

**DOI:** 10.1371/journal.pone.0031343

**Published:** 2012-02-23

**Authors:** Johan De Sutter, Nico R. Van de Veire, Sofie Struyf, Jan Philippé, Marc De Buyzere, Jo Van Damme

**Affiliations:** 1 Department of Cardiology, AZ Maria Middelares Ghent, Ghent, Belgium; 2 Heart Centre, Ghent University Hospital, Ghent, Belgium; 3 Faculty of Medicine and Pharmacy, Free University Brussels, Brussels, Belgium; 4 Laboratory of Molecular Immunology, Rega Institute, University of Leuven, Leuven, Belgium; 5 Department of Clinical Chemistry, Microbiology and Immunology, Ghent University, Ghent, Belgium; Università degli Studi di Milano, Italy

## Abstract

**Background:**

Platelet-derived chemokines are implicated in several aspects of vascular biology. However, for the chemokine platelet factor 4 variant (PF-4var/CXCL4L1), released by platelets during thrombosis and with different properties as compared to PF-4/CXCL4, its role in heart disease is not yet studied. We evaluated the determinants and prognostic value of the platelet-derived chemokines PF-4var, PF-4 and RANTES/CCL5 in patients with stable coronary artery disease (CAD).

**Methodology/Principal Findings:**

From 205 consecutive patients with stable CAD and preserved left ventricular (LV) function, blood samples were taken at inclusion and were analyzed for PF-4var, RANTES, platelet factor-4 and N-terminal pro-B-type natriuretic peptide (NT-proBNP). Patients were followed (median follow-up 2.5 years) for the combined endpoint of cardiac death, non-fatal acute myocardial infarction, stroke or hospitalization for heart failure. Independent determinants of PF-4var levels (median 10 ng/ml; interquartile range 8–16 ng/ml) were age, gender and circulating platelet number. Patients who experienced cardiac events (n = 20) during follow-up showed lower levels of PF-4var (8.5 [5.3–10] ng/ml versus 12 [8–16] ng/ml, p = 0.033). ROC analysis for events showed an area under the curve (AUC) of 0.82 (95% CI 0.73–0.90, p<0.001) for higher NT-proBNP levels and an AUC of 0.32 (95% CI 0.19–0.45, p = 0.009) for lower PF-4var levels. Cox proportional hazard analysis showed that PF-4var has an independent prognostic value on top of NT-proBNP.

**Conclusions:**

We conclude that low PF-4var/CXCL4L1 levels are associated with a poor outcome in patients with stable CAD and preserved LV function. This prognostic value is independent of NT-proBNP levels, suggesting that both neurohormonal and platelet-related factors determine outcome in these patients.

## Introduction

Platelet factor 4 (PF-4/CXCL4), the first discovered chemokine, is selectively released from stimulated platelets and has rather atypical biological properties since it is only a weak leukocyte chemoattractant compared to other chemokines. However, PF-4 is reportedly implicated in many biological processes, such as inhibition of hematopoiesis, platelet coagulation, and activation of various myeloid and lymphoid leukocyte types [1].

A striking activity of PF-4, shared with its more recently identified non-allelic variant PF-4var, is the inhibition of endothelial cell proliferation and migration [2], [3]. Angiogenesis induced by angiogenic chemokines (e.g. interleukin-8 (IL-8)/CXCL8), fibroblast growth factor (FGF) or vascular endothelial growth factor (VEGF) is significantly reduced by PF-4var and PF-4. In particular, PF-4var was found to be a more potent angiostatic chemokine than PF-4 with stronger antitumoral activity in various animal models [4].

The molecular mechanism by which PF-4 and PF-4var exert their various biological functions remains an enigma. Classical chemokines, such as IL-8/CXCL8, predominantly act through interaction with their high-affinity G protein-coupled receptors (CXCR1 and CXCR2 for IL-8/CXCL8) [5]–[7]. In addition to signaling via CXCR3 [8], [9], PF-4 also binds with high affinity to glycosaminoglycans [10] and forms heterodimers with classical growth factors, such as FGF-2 and other chemokines [11], such as RANTES/CCL5 [12]. Remarkably, PF-4var shows lower affinity for glycosaminoglycans, but shares with PF-4 the chemokine receptor CXCR3, which is also used by other angiostatic chemokines, such as interferon-induced protein-10 (IP-10/CXCL10) [13], [14]. However, these interferon-induced CXCR3 ligands are potent chemoattractants for Th1 lymphocytes and natural killer cells, whereas PF-4var rather attracts immature dendritic cells [13], [14].

Platelet-derived chemokines, including PF-4, are also implicated in several aspects of vascular biology [1], [15], such as monocyte arrest on endothelial cells (in cooperation with RANTES), induction of atherosclerotic lesions [12], promotion of thrombosis [3] and heparin-induced thrombocytopenia [16]. The role of PF-4var in processes related to atherosclerosis has, however, not yet been investigated. Therefore, the aim of the present study was to evaluate the determinants and prognostic significance of PF-4var in patients with coronary artery disease (CAD). Moreover, we compared its prognostic value to that of PF-4 and NT-proBNP, a well validated prognostic marker in patients with stable CAD [17].

## Methods

### Study populations

In order to obtain normal values for PF-4var, we evaluated 47 normal subjects. These individuals had no history of cardiovascular disease or diabetes, had no cardiac complaints and showed normal findings on a resting ECG and echocardiogram.

For the CAD patients, we prospectively evaluated 205 consecutive patients with stable CAD. The following clinical observations were considered as criteria for CAD: previous history (>6 months) of acute myocardial infarction (AMI), percutaneous coronary intervention (PCI), coronary artery bypass grafting (CABG), or documented CAD on coronary angiography (>70% stenosis). Patients with crescendo angina or angina at rest were excluded, as well as patients with recent (<6 months) acute coronary syndromes or cardiac revascularizations [18].

After overnight fasting, patients underwent a study protocol including venous blood sampling, echocardiography and evaluation of six-minutes walking distance. Echocardiography was performed with a VIVID-7 scanner (GE Vingmed Ultrasound, Horten, Norway). The left ventricular ejection fraction (LVEF) was measured in all patients using Simpson's method of discs. Patients with a LVEF≥50% were included in the present study. A six-minutes walking distance test was performed as measurement of exercise capacity. Routine blood measurements (blood cell count, creatinine) were performed according to an ISO 17025 Beltest accreditation.

### Laboratory measurements

Serum concentration of NT-proBNP was measured on an Elecsys 2010 apparatus (Roche Diagnostics, Mannheim, Germany) with an automated electrochemiluminescence sandwich immunoassay. Soluble tumour necrosis factor receptors I and II (sTNFRI and II) were measured as parameters of inflammation by ELISA (BioSource, Nivelles, Belgium) with sensitivities of 0.05 and 0.1 ng/ml, respectively.

The CC chemokine RANTES was measured using the ELISA antibody pair distributed by R&D Systems (Abingdon, UK), whereas sandwich ELISAs for the CXC chemokines PF-4 and PF-4var were developed as described [19]. Antibodies against PF-4 and PF-4var were raised in rabbits and purified by protein G chromatography. Natural PF-4 and recombinant PF-4var, purified to homogeneity in our laboratory, were used as standards. The PF-4var ELISA was specific in that PF-4 was not detectable, whereas the PF-4 ELISA measured both PF-4 and PF-4var [19].

### Follow-up

Patients were followed thereafter for the combined primary outcome measure of death due to cardiovascular causes, non-fatal myocardial infarction, non-fatal stroke or hospitalization for congestive heart failure. As secondary outcome measure the combination of MACE (death due to cardiovascular causes, non-fatal myocardial infarction, percutaneous coronary intervention or coronary artery bypass grafting) or hospitalization for congestive heart failure was evaluated. No patient was lost during the follow-up.

### Ethics

This study protocol was approved by the ethical committee of the Ghent University Hospital (Belgium) and all patients gave informed consent. The clinical investigations were conducted according to the principles of the declaration of Helsinki.

### Statistical analysis

Statistical analysis was performed using SPSS v 17.0 for Windows (SPSS Inc., 2008, Chicago, IL, USA). All continuous variables were tested for normality and log-transformed when skewed distributions were demonstrated. These are presented as mean ± standard deviation or median and interquartile range when appropriate. Differences between groups were analysed using ANOVA or Wilcoxon for continuous variables and chi-square for categorical variables. Spearman correlations were used to test the association of continuous variables with levels of PF-4var. Linear regression analysis was used to evaluate independent predictors of PF-4var levels.

For outcome analysis, multivariate analysis was performed using Cox proportional hazards models incorporating factors with predictive significance on univariate analysis and ROC analysis and hypothesized important factors.

Three models were constructed:

Model 1 clinical parameters (including age, gender, diabetes, creatinine, ejection fraction, treatment with betablockers, statins, antiplatelet drugs and ACE/ARB) with the addition of NT-proBNP.
Model 2 included the same clinical parameters as model 1, with the addition of PF-4var.
Model 3 included the same clinical parameters as model 1 with addition of NT-proBNP and PF-4var.

Cumulative event-free survival rates as a function over time were obtained by the Kaplan-Meier method. Differences in survival were analyzed by log-rank testing. Event rates per patient year follow-up were calculated.

For all analyses the level of significance (p) was set at 0.05.

## Results

### PF-4var levels and determinants in healthy control subjects

Mean age of the normal subjects was 63±11 years (range 28–79 years) and 26 were men (55%). Mean LVEF was 63±7%, mean NT-proBNP 102±83 pg/ml and mean creatinine 0.89±0.17 mg/dl. Hypertension was present in 19 individuals (40%) which required treatment with ACE inhibitors or ARB in 9 (19%), beta-blockers in 10 (21%), diuretics in 8 (17%) and calcium antagonists in 5 subjects (11%). Statins were used in 10 (21%) and aspirine in 13 individuals (28%).

Mean value of PF-4var was 14.5±7.3 (range 5–33) without significant differences between men and women (13.5±7.4 vs 15.8±7.1, p = 0.19). No significant correlations were documented between PF-4var levels and age, body mass index, LVEF, creatinine and circulating platelets. Only a weak correlation was present between PF-4var and NT-proBNP (r = −0.31, p = 0.04). Finally no significant differences in PF-4var levels were noted in subjects with and without hypertension (13.9±6.7 vs 15.2±7.9, p = 0.55) or aspirine use (16.2±8.2 vs 13.9±6.9, p = 0.37).

### Clinical characteristics of the population of CAD patients

The clinical and laboratory characteristics of the population of CAD patients and of the subgroups according to the median PF-4var value of 10 ng/ml are shown in Table 1. Patients with lower PF-4var levels were more frequently males, had a higher creatinine level and a tendency towards higher NT-proBNP levels. However, other clinical characteristics, including risk factors, previous cardiac history, medical treatment, markers of severity of heart failure and inflammation were comparable between both groups.

**Table 1:** Clinical and laboratory characteristics of the total patients population and patients with PF-4var >10 and ≤10 ng/ml.

	Total Group(n = 205)	PF-4var >10 ng/ml(n = 107)	PF-4var ≤10 ng/ml(n = 98)	p-value
Age	68±8	67±9	69±8	0.29
Gender (males)	172 (84%)	84 (79%)	89 (91%)	0.015
Previous AMI (%)	116 (57%)	60 (56%)	56 (57%)	0.89
Previous PCI (%)	71 (35%)	38 (36%)	33 (34%)	0.88
Previous CABG (%)	96 (47%)	47 (44%)	49 (50%)	0.40
Atrial fibrillation (%)	21 (10%)	10 (9%)	11 (11%)	0.82
Diabetes (%)	60 (30%)	35 (33%)	25 (26%)	0.28
Smoking (%)	22 (11%)	14 (13%)	8 (8%)	0.27
Hypertension (%)	131 (65%)	65 (62%)	66 (67%)	0.46
LVEF (%)	63±9	63±9	64±9	0.26
NYHA II-III (%)	90 (44%)	45 (42%)	45 (46%)	0.74
6 minutes WD (m)	428±115	422±118	434±113	0.51
Creatinine (mg/dl)	1±0.22	0.97±0.20	1.06±0.24	0.004
NT-proBNP (pg/ml)	164 (79–354)	137 (69–291)	206 (100–415)	0.015
sTNFRI (pg/ml)	2.99±1.15	2.96±1.26	3.03±1.02	0.67
sTNFRII (pg/ml)	8.69±3.25	8.67±3.63	8.72±2.81	0.91
Medical treatment				
Anti-platelets (%)	171 (84%)	90 (85%)	81 (82%)	0.94
Beta-blockers (%)	153 (75%)	75 (70%)	78 (80%)	0.15
ACE and/or ARB (%)	137 (67%)	73 (68%)	64 (65%)	0.41
Aldosterone antagonist (%)	14 (7%)	8 (8%)	6 (6%)	0.78
Coumarines (%)	36 (18%)	18 (17%)	18 (18%)	0.85
Statins (%)	133 (65%)	74 (69%)	59 (60%)	0.19
PF-4var (ng/ml)	10 (8–16)	15 (13–19)	7.5 (6–9)	-
PF-4 (ng/ml)	3147 (2671–3772)	3260 (2919–3962)	2961 (2381–3493)	0.001
RANTES (pg/ml)	4044 (2405–6226)	4243 (2811–6687)	3662 (1913–6034)	0.028
Platelets ×10^9^ (/l)	228±57	239±58	215±54	0.003

AMI indicates acute myocardial infarction, PCI percutaneous coronary intervention, CABG coronary artery bypass grafting, LVEF left ventricular ejection fraction, NYHA New York Heart Association, WD walking distance.

P values indicate a statistical significant difference between patients groups with PF-4var levels below and above the median value (10 ng/ml).

### Determinants of PF-4var levels in CAD patients

Significant correlations were noted between PF-4var levels and age (r = −0.15, p = 0.03), creatinine (r = −0.21, p = 0.002), NT-proBNP (r = −0.21, p = 0.003) and circulating platelet number (r = 0.233, p = 0.001). Median levels of PF-4var were significantly lower in men as compared to women (10 [7–15] ng/ml versus 14 [10–22] ng/ml, p<0.01). No significant correlations were noted between PF-4var and sTNFRI, sTNFRII, number of circulating white blood cells, lymphocytes, granulocytes or monocytes.

In multivariate regression analysis including age, gender, creatinine, NT-proBNP and circulating platelet number, levels of PF-4var were independently predicted by age (β = −0.145, p = 0.028), gender (β = −0.239, p = 0.001), and circulating platelet number (β = 0.218, p<0.001).

### Relationship between PF-4var levels and levels of PF-4 and RANTES in CAD patients

As shown in Table 1, levels of PF-4 and RANTES were significantly lower in patients with lower PF-4var levels. A modest but significant correlation was noted between PF-4var levels and PF-4 (r = 0.29, p = 0.001) but not with RANTES (r = 0.125, p = 0.074). In multivariate regression analysis including age, gender, creatinine, NT-proBNP and circulating platelet number, levels of PF-4 were independently predicted by gender (β = 0.157, p = 0.024), and circulating platelet number (β = 0.395, p<0.001). Levels of RANTES were only determined by circulating platelet number (β = 0.165, p = 0.023).

### Outcome

After a median follow-up of 2.5 years, 20 patients experienced the primary outcome measure. In patients with events, levels of NT-proBNP were higher than in patients without events (492 [267–1387] pg/ml versus 257 [76–292] pg/ml, p<0.001), whereas levels of PF-4var were lower (8.5 [5.3–10] ng/ml versus 12 [8–16] ng/ml, p = 0.033). In contrast, levels of PF-4, RANTES and platelet counts were similar in patients with and without events. ROC analysis for events showed an area under the curve (AUC) of 0.82 (95% CI 0.73–0.90, p<0.001) for higher NT-proBNP levels and an AUC of 0.32 (95% CI 0.19–0.45, p = 0.009) for lower PF-4var levels. No significant AUC was detected for PF-4, RANTES or circulating platelets.

**Figure 1:**
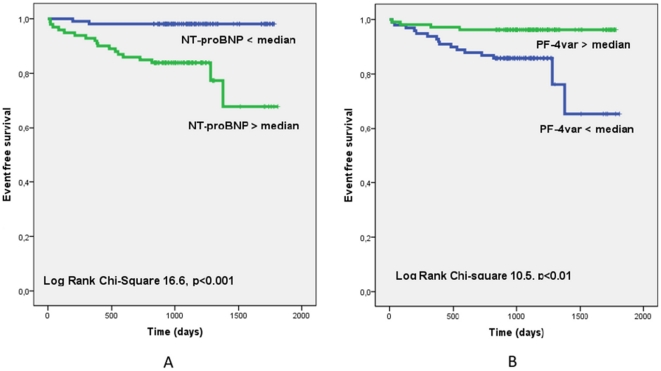
Kaplan-Meier curves for patients with levels of NT-proBNP lower and above the median value of 164 pg/ml (**figure 1A**) and Kaplan-Meier curves for patients with levels of PF-4var lower and above the median value of 10 ng/ml (**figure 1B**).

Figure 1 shows the Kaplan-Meier curves for patients (n = 205) with levels of NT-proBNP below and above the median value of 164 pg/ml (Figure 1A) and the Kaplan-Meier curves for patients (n = 205) with levels of PF-4var below and above the median value of 10 ng/ml (Figure 1B).

The results of the Cox proportional hazards models are shown in Table 2. Models 1 and 2, including clinical factors (age, gender, creatinine, LVEF, medication) and either NT-proBNP or PF-4var, showed an additional prognostic value of either NT-proBNP or PF-4var. Model 3, including clinical factors and both NT-proBNP and PF-4var, showed additional prognostic value of PF-4var on top of NT-proBNP.

**Table 2:** Multivariate analysis: hazard ratios for NT-proBNP and PF-4var.

Model	Independent predictors	Hazard ratio	CI	P-value
Model 1	Creatinine	9.95	1.18–84.23	0.038
(clinical+NT-proBNP)	Beta-blockers	0.23	0.07–0.71	0.010
	NT-proBNP	8.91	2.57–30.01	0.001
Model 2	Creatinine	18.3	12.7–98.6	0.001
(clinical+PF-4var)	Beta-blockers	0.28	0.10–0.79	0.015
	PF-4var	0.87	0.78–0.97	0.012
Model 3	Creatinine	9.24	1.03–83.12	0.047
(clinical+NT-	Beta-blockers	0.22	0.07–0.70	0.011
proBNP+PF-4var)	NT-proBNP	8.37	2.22–31.61	0.002
	PF-4var	0.89	0.79–0.99	0.036

**Figure 2:**
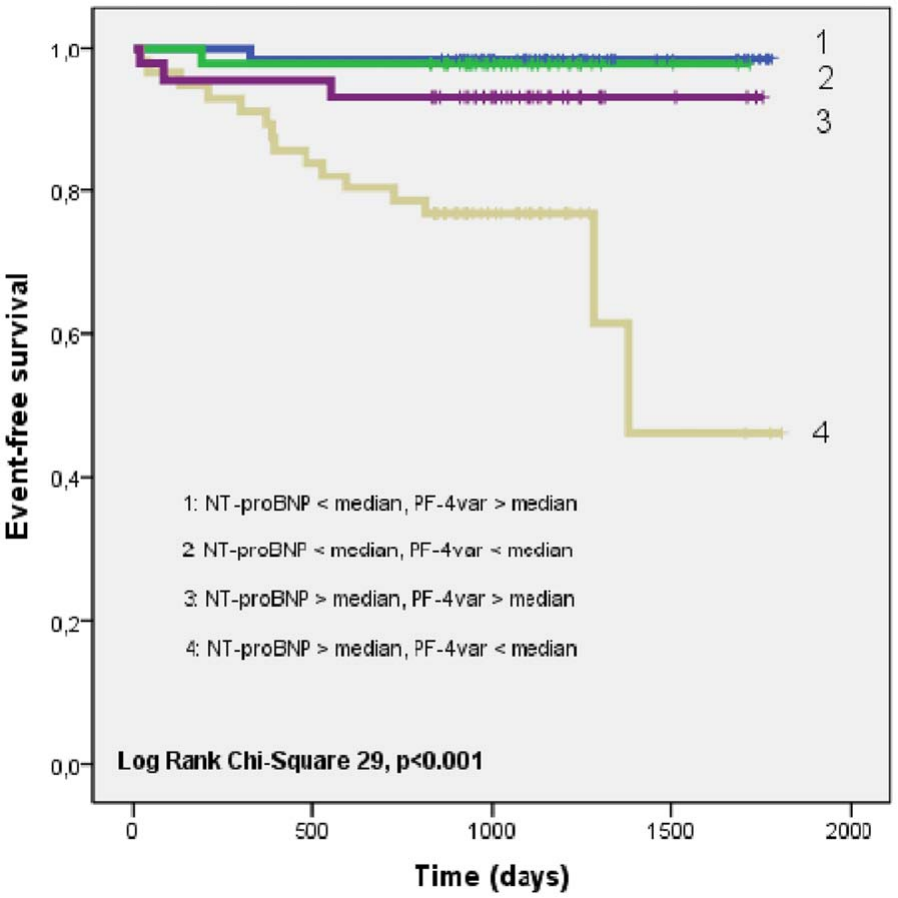
Kaplan-Meier curves showing freedom from the primary outcome measure (death due to cardiovascular causes, non-fatal myocardial infarction, non-fatal stroke or hospitalization for congestive heart failure) according to 4 groups: group 1: NT-proBNP < median and PF-4var > median (n = 64); group 2: NT-proBNP < median and PF-4var < median (n = 42); group 3: NT-proBNP > median and PF-4var > median (n = 43); group 4: NT-proBNP > median and PF-4var < median (n = 56).

Figure 2 shows the Kaplan-Meier curves for patients with, respectively, NT-proBNP levels and PF-4var levels below and above the respective median values. The group of patients with NT-proBNP levels above the median value and PF-4var levels lower than the median value showed a significantly worse outcome as compared to the other groups (p<0.001).

**Figure 3:**
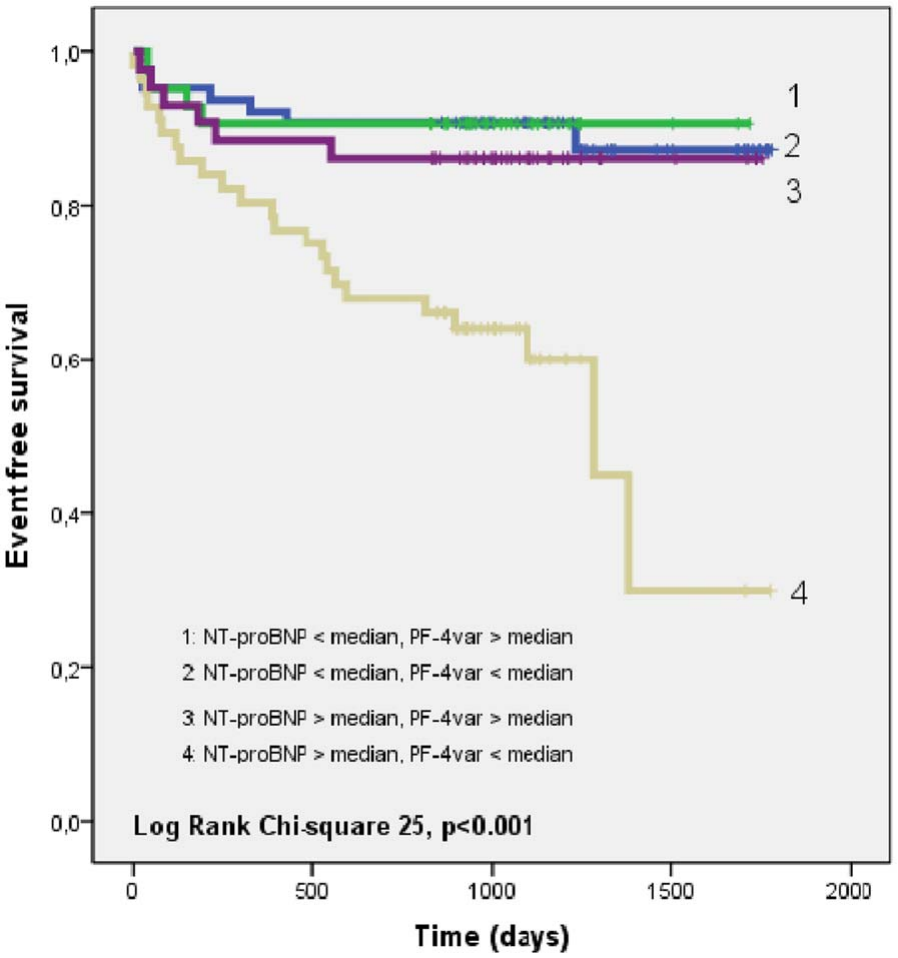
Kaplan-Meier curves showing freedom from the secondary outcome measure (death due to cardiovascular causes, non-fatal myocardial infarction, percutaneous coronary intervention, coronary artery bypass grafting or hospitalization for congestive heart failure) according to 4 groups: group 1: NT-proBNP < median and PF-4var > median (n = 64); group 2: NT-proBNP < median and PF-4var < median (n = 42); group 3: NT-proBNP > median and PF-4var > median (n = 43); group 4: NT-proBNP > median and PF-4var < median (n = 56).

The secondary outcome measure (MACE or hospitalization for congestive heart failure) was experienced by 40 patients. Figure 3 shows the Kaplan-Meier curves for patients with respectively NT-proBNP levels and PF-4var levels below and above the respective median values. Again, the group of patients with NT-proBNP levels above the median value and PF-4var levels lower than the median value showed a significantly worse outcome as compared to the other groups (p<0.001). Table 3 shows the event rates per patient year follow-up according to PF-4var and NT-proBNP levels for the primary and secondary endpoint.

**Table 3:** Event rates per patient year follow-up for the primary and secondary endpoint according to PF-4var and NT-proBNP levels.

		Primary endpoint			Secondary endpoint		
		(death due to cardiovascular causes –non fatal AMI- non fatal stroke-hospitalization congestive heart failure)			(death due to cardiovascular causes – non fatal AMI – PCI – CABG – hospitalization congestive heart failure)		
	N	Cumulative follow-up (days)	Number of events	Events/patient year FU	Cumulative follow-up (days)	Number of events	Events/patient year FU
PF-4var > median and	64	79715	1	0.0045	74213	7	0.0344
NT-proBNP < median							
PF-4var < median and	42	44907	1	0.0081	41500	4	0.0352
NT-proBNP < median							
PF-4var > median and NT-proBNP > median	43	46348	3	0.0236	43115	6	0.0508
PF-4var < median and NT-proBNP > median	56	51919	15	0.1054	45481	23	0.1846

AMI indicates acute myocardial infarction; PCI, percutaneous coronary intervention; CABG, coronary artery bypass grafting; FU, follow-up.

## Discussion

In this study we show that low levels of PF-4var, a non-allelic chemokine variant of PF-4, are associated with a worse outcome in patients with stable CAD and preserved left ventricular function. Moreover, PF-4var showed additional prognostic value on top of NT-proBNP, a well validated prognostic parameter in CAD patients. In contrast, levels of the other platelet-derived chemokines, PF-4 and RANTES, were not associated with outcome.

Chemokines constitute a large family of chemotactic cytokines which stimulate migration of various leucocyte subsets during normal and pathological conditions [5]–[7]. PF-4var structurally differs from PF-4 in only 3 amino acids. PF-4var and PF-4 are stored in the α-granules of platelets, together with some other chemokines such as platelet basic protein (PBP/CXCL7) and RANTES. PF-4var and PF-4 are released upon platelet activation by e.g. thrombin, a much lower amount of PF-4var being secreted. PF-4var and PF-4 are inducible in monocytes stimulated by inflammatory mediators [19]. Furthermore, PF-4var but not PF-4 is produced constitutively by smooth muscle cells and by sarcoma cells after induction with cytokines, such as IL-1β and TNF-α [19], [20], indicating that the regulated expression of these two PF-4 variants is different.

At high concentration (>1 µg/ml) PF-4 inhibits megakaryopoiesis and functions as negative autocrine regulator *in vitro* [21] and *in vivo* [22]. Further, PF-4 has pro-thrombotic activity by binding heparin, but it can also function as an anti-coagulant via the generation of activated protein C [23]. However, in PF-4 knock-out mice, occlusive thrombi developed more slowly, whereas PF-4 infusion restored thrombus formation, indicating that this chemokine is rather pro-thrombotic [3]. In view of the high concentrations required for these effects, it remains to be seen whether PF-4var released into the circulation at much lower concentrations is implicated in coagulation and thrombogenesis.

PF-4 does not attract monocytes [19], but promotes monocyte survival and differentiation into specific subtypes of macrophages [24]–[26] and dendritic cells with altered cytokine producing capacities [27]. However, PF-4 stimulates monocytes to induce apoptosis in endothelial cells [28]. Although PF-4var is also not chemotactic for monocytes, it does stimulate the migration of immature dendritic cells, activated T cells and NK cells [13].

Despite its lower affinity for heparin, PF-4var is a more potent inhibitor of endothelial cell growth and migration than PF-4, by modulating the mitogenic activity of growth factors (e.g. basic fibroblast growth factor) or antagonizing the chemotactic activity of angiogenic chemokines (e.g. IL-8/CXCL8) [2]. The possible mechanisms to explain the angiostatic effect of PF-4var include interaction with CXCR3, displacement of growth factors for binding to glycosaminoglycans, and direct interference with angiogenic growth factors and chemokine heteromultimerization. Although the formation of such chemokine heteromultimers [11] inhibits endothelial cell growth, the disruption of the proinflammatory interaction between the platelet-derived chemokines PF-4 and RANTES also alters monocyte recruitment and inhibits atherosclerosis [12]. Therefore, it needs to be investigated in depth whether PF-4var, showing physico-chemical and biological properties different from PF-4, can affect atherosclerosis and why reduced levels of PF-4var are associated with a poor outcome in CAD patients. Also, the reasons of lower PF-4var levels (e.g. lower production or higher degradation of PF-4var) in CAD patients with an adverse outcome need further study. Although there is no mechanistic evidence about the involvement of PF-4var in CAD, one may wonder whether low PF-4var blood levels could reflect changes in an ongoing chemokine response. However, the PF-4var levels did not correlate with those of the inflammatory chemokine RANTES. Alternatively, it could be hypothesized that the lowered PF-4var levels reflect the impact of blood platelet counts on CAD. However, not only PF-4var, but also PF-4 and RANTES levels correlated with the number of circulating platelets, indicating that this correlation is not PF-4var specific. Further, PF-4var but not PF-4 levels are associated with CAD outcome, which is in line with the fact that both CXCR3 ligands differ in biological potency as angiostatic factors and show different affinity for glycosaminoglycans. In contrast to the platelet-derived PF-4 and PF-4var, the other CXCR3 ligands are not expected to occur at detectable levels in the blood circulation of healthy persons. We verified the presence of CXCL11/I-TAC in serum from both control (n = 25) and CAD patients (n = 42), but found indeed that no CXCL11/I-TAC was detectable (all levels remained below 80 pg/ml). Thus, it can be concluded that the CXCR3 ligand PF-4var is a selective marker for CAD.

Importantly, our results show that the prognostic value of PF-4var is independent of NT-proBNP levels in stable CAD patients. This indicates that low PF-4var levels affect outcome in a different way as compared to neurohormonal activation. Indeed, unlike NT-proBNP, no significant PF-4var gene expression has been detected in heart and brain (NCBI Gene Expression Omnibus database). However, the NT-proBNP receptor NPR-A is most abundantly expressed in large blood vessels, and endothelial cells are the major target cells for angiostatic PF-4var via binding to CXCR3. Of note, no correlation was found between PF-4var levels and markers of inflammation such as sTNFRI and sTNFRII or the number of circulating white blood cells, lymphocytes, granulocytes or monocytes. This is in contrast with the well known relationship between NT-proBNP levels and different inflammatory markers including high-sensitive C-reactive protein [29]. Remarkably, patients who presented with both low PF-4var levels and high NT-proBNP levels, had a clear adverse outcome as compared to the other groups. This was not only noted for the primary outcome measurement, but even more for the secondary outcome measurement which included revascularization during follow-up. These observations implicate that an approach with two different biomarkers, probably representing distinct pathophysiological pathways (one evaluating cardiovascular function and remodeling and one evaluating platelet function) may be relevant for risk stratification of patients with stable CAD. A recent paper by Schnabel et al. [30] looked at a multiple marker approach to risk stratification in a large population of patients with stable CAD. Although the different markers (reflecting inflammation, lipid metabolism, renal function and cardiovascular function and remodeling) offered incremental predictive ability over established risk factors, combination of the single markers did not enhance risk prediction from a clinical perspective. Markers of platelet function and activation were however not included in that study. Also, the clear prognostic impact of low PF-4var levels may be further evaluated as potential parameter for new anti-platelet drug therapies in patients with stable CAD.

In conclusion, low levels of the chemokine PF-4var, a non-allelic variant of PF-4, are associated with a poor outcome in patients with stable CAD. The prognostic value of PF-4var is independent of the prognostic value of NT-proBNP, a well validated prognostic marker in these patients. These results warrant further studies regarding the pathophysiological function of PF-4var and its potential role as marker of platelet-related activation in patients with stable CAD.
